# Wolfram Syndrome: A Curious Case of Repetitive Loss of Consciousness

**DOI:** 10.7759/cureus.46426

**Published:** 2023-10-03

**Authors:** Margarida M Carvalho, Rafael Jesus, Ana Mendes, Pedro Guimarães, Bebiana Conde

**Affiliations:** 1 Pulmonology, Tras-os-Montes and Alto Douro Hospital Centre, Vila Real, PRT; 2 Neurology, Tras-os-Montes and Alto Douro Hospital Centre, Vila Real, PRT; 3 Neurophysiology, Tras-os-Montes and Alto Douro Hospital Centre, Vila Real, PRT

**Keywords:** non invasive mechanical ventilation, bulbar dysfunction, brain stem atrophy, obstructive sleep apnea, central apnea, wolfram syndrome

## Abstract

Wolfram syndrome is a rare, multisystemic, progressive, and autosomal-recessive genetic disease, characterized by diabetes mellitus and diabetes insipidus, optic nerve atrophy, deafness, and other neurological signs. The diagnosis is usually based on history and clinical manifestations but genetic tests are necessary for confirmation. Currently, there are no treatments available to cure or delay disease progression. This report describes a case of a 23-year-old male diagnosed with Wolfram syndrome who presented to the emergency department with several episodes of loss of consciousness. This case reinforces the need for an early diagnosis of obstructive and central apneas, respiratory failure, and dysphagia, in order to prevent and treat the complications of this disease and to improve patients’ quality of life.

## Introduction

Wolfram syndrome (WS) is a multisystemic progressive and autosomal recessive genetic disease, whose main clinical features are diabetes insipidus, diabetes mellitus(DM), optic nerve atrophy, deafness, and urinary tract problems [1-5]. It affects about one in 770,000 people around the world and, therefore, is considered a rare disease [2,4]. WS has a higher prevalence reported in Lebanon, where it was found that in 399 patients with juvenile-onset diabetes, and 22 had a genetically confirmed diagnosis of WS type 1 [1,3,6]. No epidemiological data are available regarding WS type 2 due to patients not having been diagnosed in all countries [3].

Studies have identified two genes responsible for WS: Wolfram Syndrome 1 (*WFS1*) and Wolfram Syndrome 2 (*WFS2*) [5,7]. The *WFS1 *encodes a transmembrane glycoprotein in the endoplasmic reticulum (ER) named wolframin that serves as a calcium channel [1,3,5]. This suggests that wolframin may play a role in ER homeostasis [1,3,5]. There is high expression of *WFS1* in the lung, heart, brain, pancreatic β-cells, and placenta [1,3,5]. Autosomal recessive mutations in this gene cause the classic form of WS [1].

WS type 2, a rare and neurodegenerative type of WS, is caused by mutations in the CDGSH iron-sulfur domain-containing protein 2 (*CIDS2*) gene [1,5]. *CISD2 *encodes the protein named small intermembrane endoplasmic reticulum protein, which has a central role in the exchanges between endoplasmic reticulum and mitochondria, in the regulation of mitochondrial homeostasis, and in the activation of apoptosis and autophagy [1,8]. This protein is highly expressed in brain and pancreas tissues [1]. Patients diagnosed with WS type 2 presented with bleeding, defective platelet aggregation, upper intestinal ulcer, and an absence of diabetes insipidusand psychiatric disorders [1,3].

Lately, there have been reports of dominant disease with or without DM, atypical forms of disease related to one or two mutations in *WFS1* [5]. Currently, there are no treatments able to cure or delay the progression of this disease [3]. Therefore, it is essential to offer appropriate supportive care, preventing and treating early complications, in order to improve these patients’ quality of life [5].

In this case report, we describe a case of a patient with WS that presented to the Emergency Department (ED) due to several episodes of loss of consciousness. Loss of consciousness is one of the most common reasons for admission to the ED. It is essential to make a detailed characterization of the episode to discriminate between differential diagnoses [2].

This article was previously presented as a meeting poster at the World Sleep 2022 on March 14, 2022.

## Case presentation

We report the case of a male patient diagnosed with diabetes mellitus at the age of six years old. When the patient was eight years old, he was also diagnosed with diabetes insipidus, cognitive development delay (intelligence quotient 70), hypersomnolence, bilateral optic nerve atrophy, primary hypogonadism with testicular atrophy, neurogenic bladder, and hydroureteronephrosis. At that time, he was medicated with insulin glargine, insulin glulisine, oxybutynin, tamsulosin, and desmopressin. With the suspicion of WS, a genetic test was made, which identified the following homozygous variants in the *WFS1* gene: c.482G>A (p.Arg161GIn) in exon 5, and c.1066T>C (p.Ser356Pro) in exon 8. The family members were asymptomatic and their genetic study was negative.

**Figure 1:**
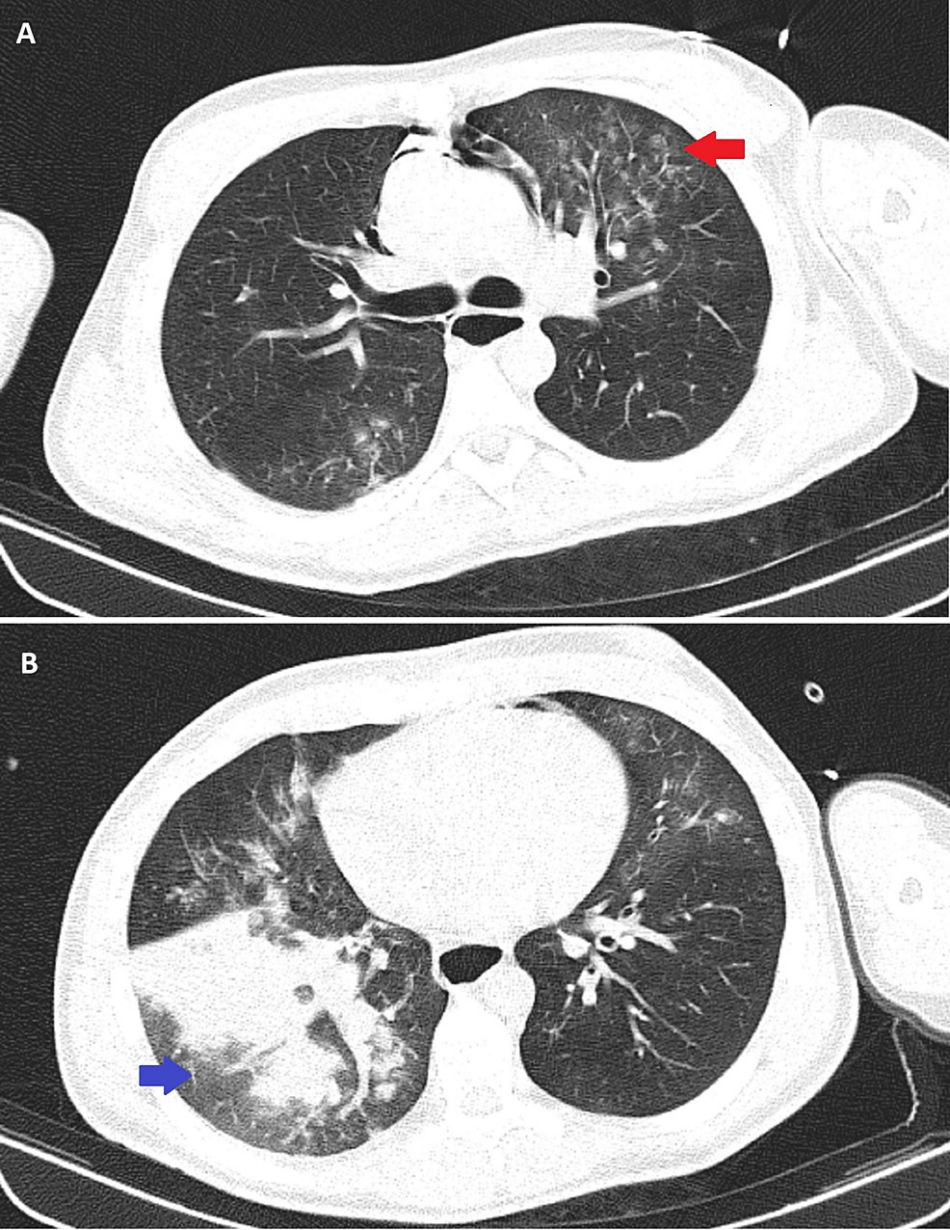
Chest computed tomography showing: (A) pulmonary infiltration in the left superior lobe (red arrow); (B) a consolidation in the right inferior lobe (blue arrow).

**Figure 2:**
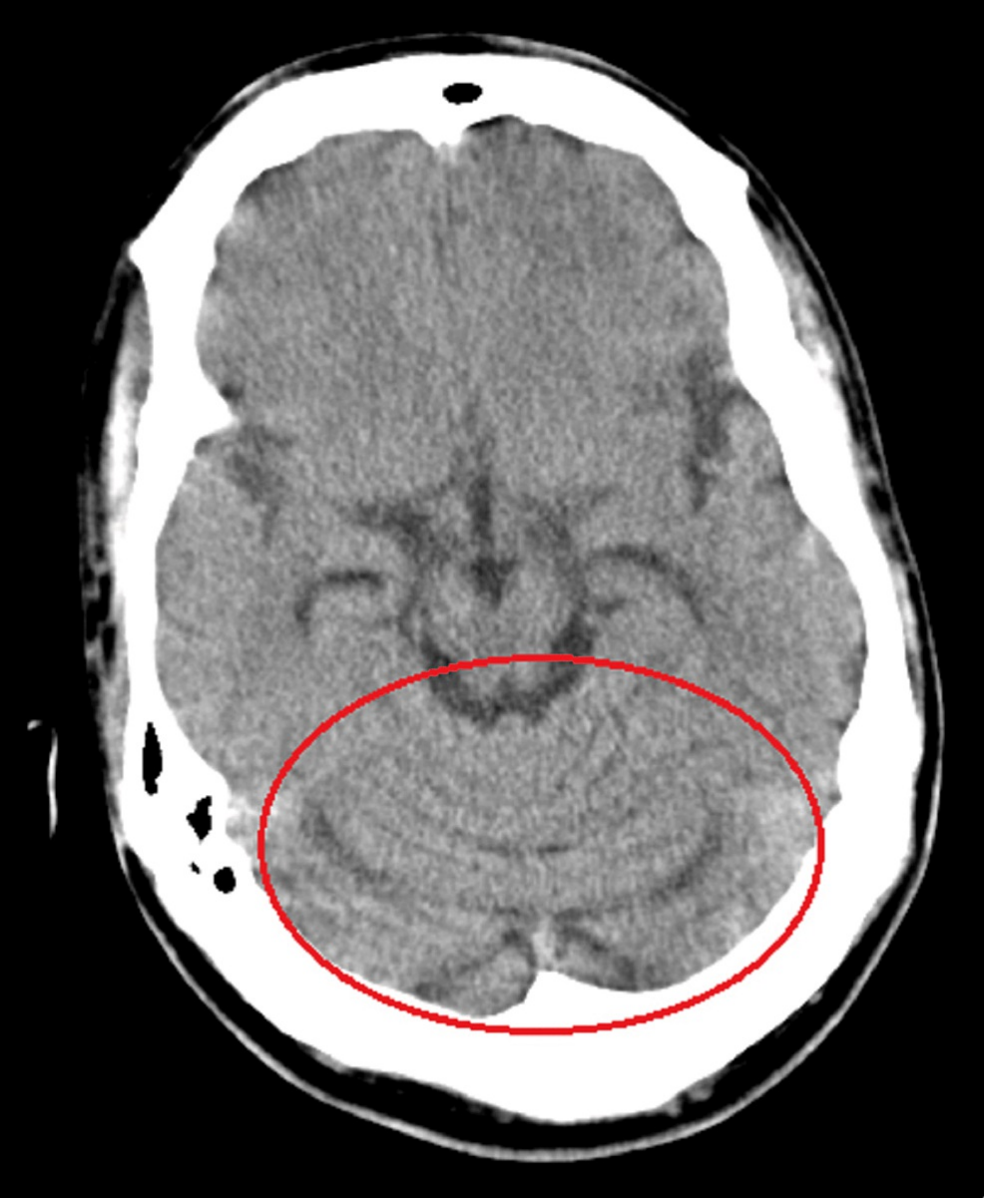
Brain computed tomography revealing cerebellar atrophy (red circle).

When he was 23 years old, the patient was admitted to the ED with a three-week-long dysphagia for solids and liquids and several episodes of loss of consciousness. These episodes occurred several times during the permanence in the ED and they were accompanied by ocular retroversion, trunk stiffness, and short-lasting distal limb myoclonias. All of the episodes were associated with lip cyanosis, paradoxical breathing followed by absence of respiratory movements, and severe hypoxemia, with apparent superior airway obstruction. The patient recovered spontaneously after approximately two minutes. Blood analysis and electrocardiogram were normal. Blood gas analysis, carried out with supplemental oxygen (fraction of inspired oxygen (FiO2) of 40%), showed type 2 respiratory insufficiency - pH 7.35, partial pressure of oxygen (PaO2) 65mmHg, partial pressure of carbon dioxide (PaCO2) 50mmHg, serum bicarbonate (HCO3) 27.4mmol/L, arterial oxygen saturation (SaO2) 91%. Chest computed tomography (CT) showed a pulmonary infiltration in the left superior lobe and a consolidation in the right inferior lobe (Figure 1). A brain CT scan revealed cerebellar atrophy (Figure 2). Shortly after one of the episodes, he was submitted to an electroencephalogram, which revealed no abnormalities including of the epileptiform type.

After a multidisciplinary discussion between pulmonology, neurology, and intensive care, the patient was admitted to the intensive care unit and empiric antibiotic treatment was initiated for bilateral pneumonia and non-invasive mechanical ventilation (NIMV), bilevel positive airway pressure (BPAP) spontaneous timed (ST) mode, with expiratory positive airway pressure (EPAP) of 7cmH2O and inspiratory positive airway pressure (IPAP) of 15cmH2O, respiratory frequency of 13cpm, with FiO2 45% and oronasal mask. In the first four days, the NIVM was maintained continuously and after that, the patient was under NIMV only at night.

**Figure 3:**
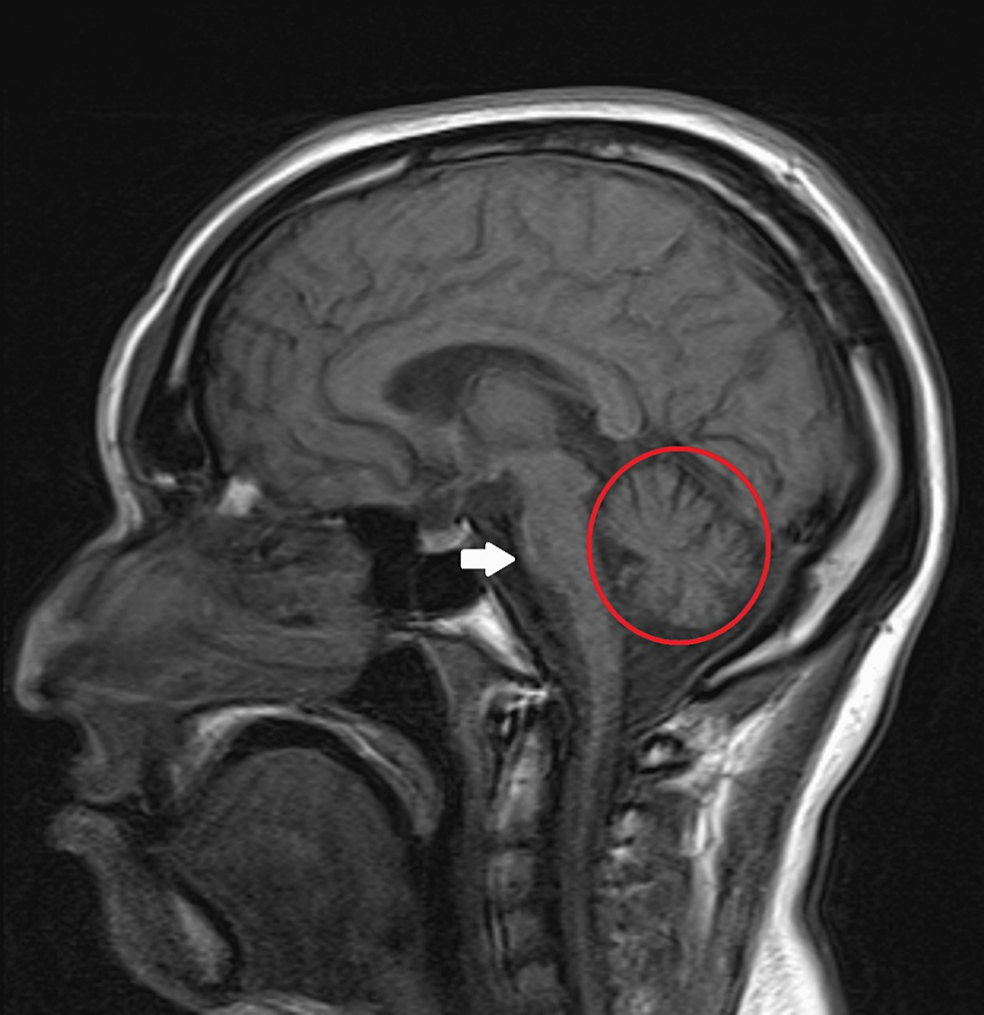
Brain magnetic resonance imaging revealed cerebellar atrophy, with widening of the sulci of the cerebellar hemispheres (red circle) and brain stem atrophy (white arrow).

Brain magnetic resonance imaging (MRI) (Figure 3) showed cerebellar atrophy with widening of the sulci of the cerebellar hemispheres and brain stem atrophy. No other changes in signal evolution or brain appearance morphology are apparent, nor any restrictions on the diffusibility of water molecules. The high convexity sulci are patent and the cerebellar tonsils are normally positioned.

**Figure 4:**
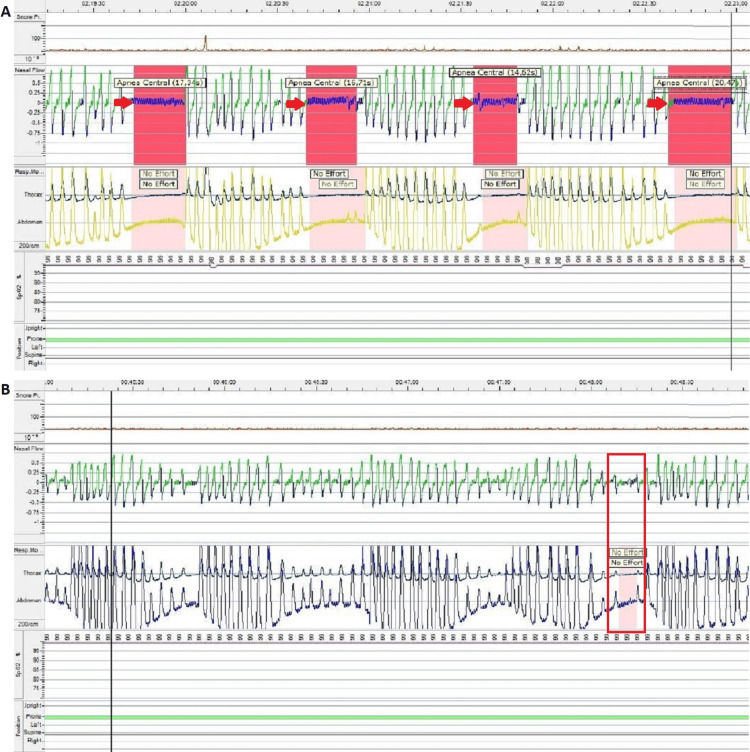
Type III sleep study showing: (A) several central apneas (red arrows); (B) central hypopnea (red rectangle).

During hospitalization, the patient was evaluated by a doctor of physical medicine and rehabilitation who confirmed the dysphagia for solids and liquids and a percutaneous endoscopic gastrostomy was performed. The patient was also submitted to a type III sleep study, with supplemental oxygen (Figure 4). A mild obstructive sleep apnea (OSA) was diagnosed, with an apnea-hypopnea index (AHI) of 9.1/h. Central events were also recorded, with a central apnea index (CAI) of 8.1/h.

**Figure 5:**
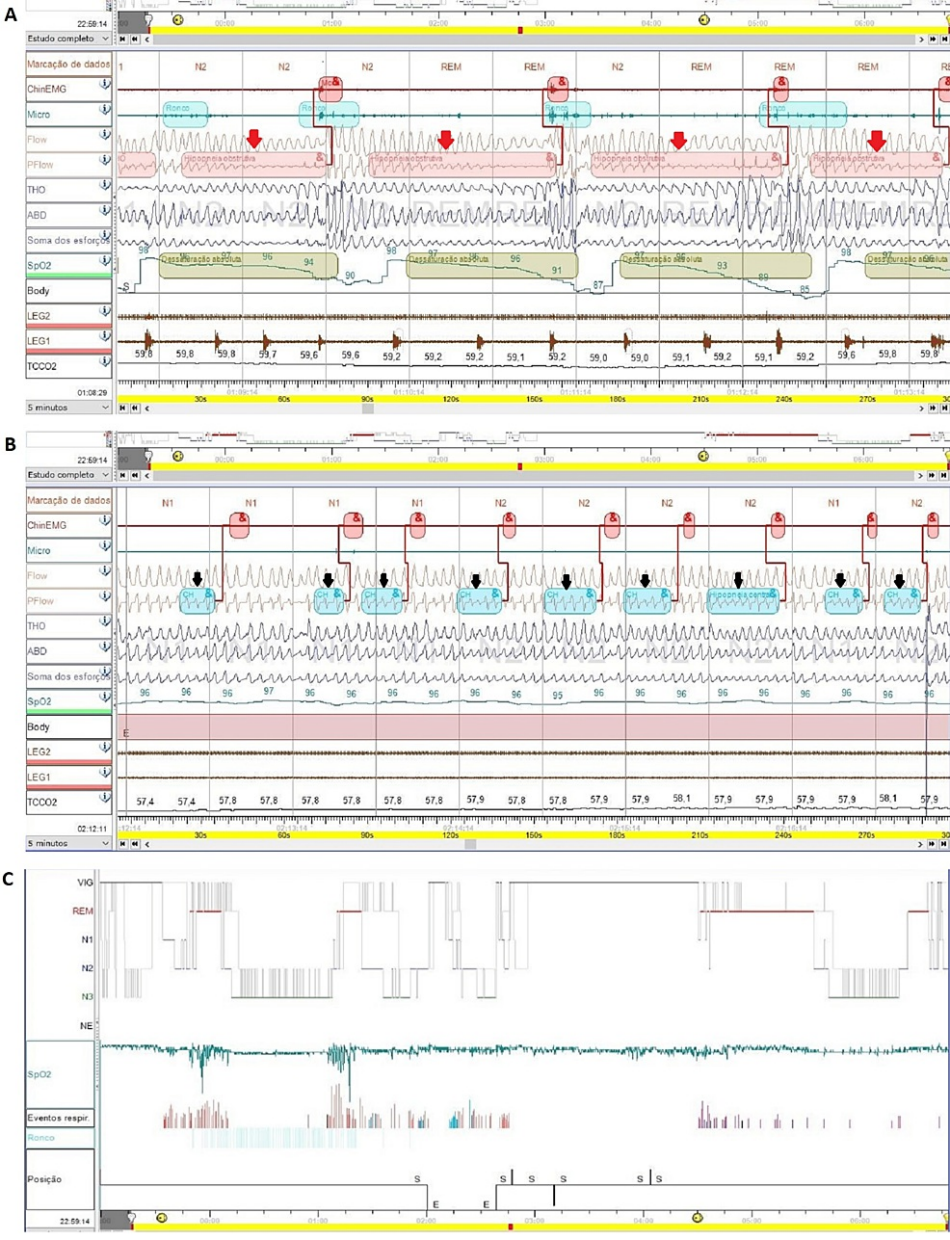
Split-night polysomnography showing: (A) obstructive hypopneas in the diagnostic portion of laboratory polysomnography (red arrows); (B) central hypopneas in the diagnostic portion of laboratory polysomnography (black arrows); (C) hypnogram of split-night polysomnography.

After medical discharge, in order to better clarify the clinical case, split-night polysomnography was performed (Figure 5) a month and a half after the type III sleep study. It showed decreased sleep efficiency for his age group (69.2% of total study time), with altered sleep patterns and mid-nocturnal insomnia (compatible with the moment of placement of NIMV). The study registered one Sleep Onset REM Period (SOREMP), respiratory disturbance index (RDI) of 39.4/h, and CAI of 0.3/h. It also registered a minimum peripheral oxygen saturation (SpO2) of 71% (associated with respiratory events), an average SpO2 of 96%, a percentage of time with a SpO2 < 90% (CT90) of 1.6%, and an average cutaneous carbon dioxide tension of 59mmHg.

Therefore, the polysomnography was compatible with the diagnosis of severe obstructive sleep apnea and hypoventilation during sleep. The study confirmed that correction of respiratory events and oxygen saturation level was obtained with the application of NIMV in BPAP ST mode, with EPAP of 8cmH2O and IPAP of 15cmH2O, respiratory frequency of 14cpm, minimum inspiratory time of half a second, and maximum inspiratory time of two seconds. There was no need for supplemental oxygen and a good adaptation was obtained to the ventilatory mode, to the established pressures, and to the oronasal interface.

During reassessment at the consultation after medical discharge, the patient presented good adherence to the ventilator with effective correction of respiratory events. He improved considerably since the initiation of NIMV. In two years of follow-up, the patient maintained adherence to NIMV, with effective correction of respiratory events. Nocturnal oximetry, performed without supplemental oxygen, revealed an average SpO2 of 98%. The patient had good symptomatic control, denied dyspnea, and there were no other episodes of hospitalization due to respiratory pathology or new episodes of loss of consciousness.

## Discussion

WS, sometimes referred to as DIDMOAD (diabetes insipidus, diabetes mellitus, optic atrophy, and deafness) due to its main clinical features as an autosomal-recessive genetic disorder, was first described in 1938 by Wolfram and Wagener [1,4]. They described four siblings born from consanguineous parents who were diagnosed with DM and optic atrophy [3,8]. The main clinical features of WS include DM, central diabetes insipidus, optic nerve atrophy, sensorineural deafness, and other neurologic disabilities and urinary tract problems [2,3,5].

DMis often diagnosed around six years old [2]. It is often milder than type 1 DM, with low insulin requirement and rare diabetic ketoacidosis episodes and microvascular complications [3]. However, hypoglycemia episodes may be frequent due to neurological dysfunctions [3]. Optic nerve atrophy, usually diagnosed before 15 years old, is characterized by a progressive decrease in visual acuity, loss of peripheral vision, and a color vision defect, which leads to blindness [2,3,5,9]. Central diabetes insipidus, more prevalent in the second decade of life, affects 70% of these patients [2,3]. It manifests as polyuria due to the decreased capacity to concentrate urine [3]. Around two-thirds of WS patients develop hearing loss that can manifest either as congenital deafness or as a slight hearing impairment which, sometimes, worsens over time, due to central nervous system degenerative process [2,3]. Neurological manifestations are common and frequently occur later in life, usually in the third and fourth decades [2,5]. They progress until brain atrophy, more noticeable in the pons, cerebellum, and medulla [3]. Although cerebellar ataxia is the most frequent neurologic complication, brain stem atrophy leads to central apneas that can result in death [2,5]. Other neurological manifestations are epilepsy, nystagmus, and dysphagia [5]. Urinary tract problems affect approximately 60-90% of these patients [2]. Urinary incontinence and neurogenic bladder with hydroureter are the most common and usually are due to neurological impairment [3,5,10]. An additional abnormality in WS is hypogonadism, which is more frequent in male patients [3,5,10].

The diagnosis of DMand optic nerve atrophy before 16 years old raises the suspicion of this diagnosis [1,2,5]. Furthermore, the concomitant diagnosis of diabetes insipidus, neurological signs including ataxia and epilepsy, neurogenic bladder, and sensorineural hearing loss increases the likelihood of this diagnosis [2]. However, genetic tests are necessary to confirm it [2,5]. Early diagnosis of WS type 1 is imperative to prevent complications [1,5]. Family members should be guided towards genetic counseling and genetic tests such as exome sequencing and genome sequencing-based diagnostic methods, even if they are asymptomatic [1,5]. Differential diagnoses should be made with mitochondrial disorders, autosomal dominant optic nerve atrophy, mutant *WFS1* gene-induced deafness, Friedreich ataxia, Alström syndrome, and Bardet-Biedl syndrome [1,2,11].

The main goals for WS treatment are stopping the progression of the disease and replacing damaged tissues [5,7]. Some investigational drugs (4-phenyl butyric acid, tauroursodeoxycholic acid, and dantrolene) target ER and can prevent the death of neurons and β-cells in these patients [12,13]. A better understanding of the pathogenesis is needed in order to develop novel therapeutics for this disease [12,13]. Currently, there is no specific and effective pharmacological therapy available, but a careful clinical follow-up, treatment of each disorder associated with WS, and supportive care can improve patients’ quality of life, relieving severe and progressive symptoms [3,5,7].

Patients with WS have a poor prognosis [1,2]. Most patients die prematurely, with the median age of death being 30 years [1,2]. This can be explained by the rapidly progressive clinical course of this disease, with brain stem atrophy and bulbar dysfunction appearing early in life [1-3,5,14]. This leads to respiratory failure and dysphagia, which can cause aspiration pneumonia, which is one of the most frequent causes of mortality [1-3,5,14].

## Conclusions

This case reports a patient with WS1, a rare disorder that manifests early in life, progressing with brain stem atrophy, bulbar dysfunction, and respiratory failure. Although the disease can cause seizures, other manifestations such as obstructive and central apneas are more common. The early diagnosis of the disease and the treatment with NIMV in patients with respiratory failure or with central and obstructive apneas is essential.

Furthermore, it is essential to assess swallowing capacity for early detection of dysphagia and placement of endoscopic percutaneous gastrostomy, to prevent episodes of aspiration pneumonia, one of the main causes of mortality. This case reinforces that it is crucial to have a multidisciplinary approach in order to be able to prevent and treat the complications of this disease and to try to maintain the best possible quality of life for such patients.

## References

[1] Rigoli L, Caruso V, Salzano G, Lombardo F (2022). Wolfram syndrome 1: from genetics to therapy. Int J Environ Res Public Health.

[2] Urano F (2016). Wolfram syndrome: diagnosis, management, and treatment. Curr Diab Rep.

[3] Delvecchio M, Iacoviello M, Pantaleo A, Resta N (2021). Clinical spectrum associated with wolfram syndrome type 1 and type 2: a review on genotype-phenotype correlations. Int J Environ Res Public Health.

[4] Ferreras C, Gorito V, Pedro J, Ferreira S, Costa C, Santos Silva R, Castro Correia C (2021). Wolfram syndrome: Portuguese research. Endokrynol Pol.

[5] Pallotta MT, Tascini G, Crispoldi R, Orabona C, Mondanelli G, Grohmann U, Esposito S (2019). Wolfram syndrome, a rare neurodegenerative disease: from pathogenesis to future treatment perspectives. J Transl Med.

[6] Zalloua PA, Azar ST, Delépine M (2008). WFS1 mutations are frequent monogenic causes of juvenile-onset diabetes mellitus in Lebanon. Hum Mol Genet.

[7] Delprat B, Maurice T, Delettre C (2018). Wolfram syndrome: MAMs' connection?. Cell Death Dis.

[8] Rosanio FM, Di Candia F, Occhiati L (2022). Wolfram syndrome type 2: a systematic review of a not easily identifiable clinical spectrum. Int J Environ Res Public Health.

[9] Megighian D, Savastano M (2004). Wolfram syndrome. Int J Pediatr Otorhinolaryngol.

[10] Fabbri LP, Nucera M, Grippo A, Menicucci A, De Feo ML, Beechi C, Al Malyan M (2005). Wolfram syndrome. How much could knowledge challenge the fate? A case report. Med Sci Monit.

[11] Barrett TG, Bundey SE (1997). Wolfram (DIDMOAD) syndrome. J Med Genet.

[12] Kumar J, Ahmed A, Khan M, Ahmed Y (2023). There’s more than meets the eye: Wolfram syndrome in a type I diabetic patient. J Med Cases.

[13] Shang L, Hua H, Foo K (2014). β-cell dysfunction due to increased ER stress in a stem cell model of Wolfram syndrome. Diabetes.

[14] Iafusco D, Zanfardino A, Piscopo A (2022). Metabolic treatment of Wolfram syndrome. Int J Environ Res Public Health.

